# CDK4/6 and BET inhibitors synergistically suppress pancreatic tumor growth and epithelial-to-mesenchymal transition by regulating the GSK3β-mediated Wnt/β-catenin pathway

**DOI:** 10.20517/cdr.2025.38

**Published:** 2025-09-26

**Authors:** Jiangning Gu, Zihao Dai, Tianci Shen, Xiang Chen, Zhuo Yang, Shibo Sun, Dan Chen, Haifeng Luo, Xiuli Wang, Jianqiang Xu

**Affiliations:** ^1^Department of Endoscope, General Hospital of Northern Theater Command, Shenyang 110011, Liaoning, China.; ^2^Department of Hepatobiliary Surgery, the First Affiliated Hospital of Dalian Medical University, Dalian 116011, Liaoning, China.; ^3^School of Chemical Engineering, Ocean Technology and Life Science (CEOTLS) & Panjin Institute of Industrial Technology, Dalian University of Technology, Panjin 124221, Liaoning, China.; ^4^Department of Pathology, the First Affiliated Hospital of Dalian Medical University, Dalian 116011, Liaoning, China.; ^5^College of Basic Medical Sciences, Dalian Medical University, Dalian 116044, Liaoning, China.; ^#^Authors contributed equally.

**Keywords:** Pancreatic cancer, pancreatic ductal adenocarcinoma, epithelial-to-mesenchymal transition, CDK4/6 inhibitor PD-0332991, BET inhibitor JQ1, Wnt/β-catenin pathway

## Abstract

**Aim:** Cyclin-dependent kinases 4 and 6 (CDK4/6) are frequently upregulated in pancreatic ductal adenocarcinoma (PDAC) and are associated with poor overall survival. Although CDK4/6 inhibition suppresses tumor cell proliferation, it paradoxically promotes metastasis and invasion, and the mechanisms underlying this effect remain unclear.

**Methods:** We evaluated the effects of the CDK4/6 inhibitor palbociclib (PD-0332991) and the bromodomain and extra-terminal (BET) inhibitor JQ1, administered individually and in combination, on human PDAC cell lines *in vitro* and on tumor growth in an orthotopic mouse model.

**Results:** Palbociclib modestly inhibited pancreatic tumor growth but significantly enhanced tumor cell migration, invasion, and epithelial-to-mesenchymal transition (EMT). In contrast, co-treatment with JQ1 potentiated palbociclib’s anti-proliferative effects and reversed EMT. Mechanistically, CDK4/6 inhibition activated the canonical Wnt/β-catenin pathway via Ser9 phosphorylation of GSK3β, whereas BET inhibition disrupted the cross-talk between Wnt/β-catenin and TGF-β/Smad signaling. Combined inhibition of CDK4/6 and BET produced a synergistic antitumor effect *in vitro* and *in vivo*.

**Conclusion:** Our findings support a combined therapeutic strategy targeting CDK4/6 and BET proteins to achieve synergistic inhibition of PDAC progression.

## INTRODUCTION

Pancreatic cancer, particularly pancreatic ductal adenocarcinoma (PDAC), remains one of the most lethal solid malignancies worldwide^[1]^. Fewer than 8% of patients survive five years, and only about 20% are candidates for curative resection. Unlike other solid tumors such as breast, non-small-cell lung, and prostate cancers, PDAC relies almost exclusively on chemotherapy, since effective molecular targets for targeted or immune-based therapies have not yet been identified^[2,3]^.

The most frequent genetic alteration in PDAC involves the Kirsten rat sarcoma viral oncogene homolog (KRAS)^[4]^, which activates multiple oncogenic cascades - including RAF/MEK/ERK, PI3K/AKT, and nuclear factor-κB (NF-κB) - to drive malignant phenotypes^[5,6]^. AMG-510, the first clinically approved KRAS inhibitor^[7]^, selectively targets KRAS^G12C^. However, the G12D and G12V variants predominate in PDAC, and clinical data on AMG-510 in PDAC remain limited (NCT03600883). Loss-of-function mutations in CDKN2A occur in up to 90% of PDAC cases^[8-11]^, resulting in p16^INK4a^ inactivation^[12]^, deregulation of cyclin-dependent kinases 4 and 6 (CDK4/6)–Cyclin D complexes, and retinoblastoma (RB) protein phosphorylation, thereby promoting tumor cell proliferation.

Notably, CDK4/6 is frequently upregulated across diverse human cancers^[13,14]^ and has emerged as a promising therapeutic target^[15-18]^. Three CDK4/6 inhibitors - palbociclib (PD-0332991)^[19]^, abemaciclib (LY2835219)^[20]^, and ribociclib (LEE011)^[21]^ - have received the Food and Drug Administration (FDA) approval for ER-positive breast cancer, substantially improving overall and progression-free survival^[22]^. However, when administered as monotherapy in other solid tumors, these agents have shown limited efficacy (e.g., NCT02693535), likely reflecting compensatory signaling networks, activation of alternative oncogenic pathways, or emergence of secondary resistance mutations^[23,24]^.

To overcome resistance, various combination approaches have been explored at the preclinical level, including inhibitors of PI3K/AKT/mTOR, MEK, and PARP^[25,26]^. For example, SGK3 inhibition has been reported to potentiate palbociclib’s antitumor activity in cervical cancer models^[27]^, and trametinib plus CDK4/6 inhibition has been trialed as third-line therapy in metastatic PDAC^[28]^. These findings underscore the importance of dissecting drug-induced network rewiring to guide rational combination regimens.

Bromodomain and extra-terminal (BET) inhibitors, such as the small molecule JQ1 (an investigational agent in clinical trials for PDAC and other malignancies)^[29]^, modulate gene expression through epigenetic mechanisms, including histone acetylation and deacetylation^[30,31]^. BET inhibition by JQ1, alone or in combination with histone deacetylase (HDAC) inhibitors such as suberanilohydroxamic acid (SAHA), has been shown to suppress PDAC growth and progression *in vitro* and *in vivo*^[32]^. JQ1 can also decrease HMGCS2 expression, sensitizing resistant pancreatic tumors to gemcitabine^[33]^, and novel CAF-targeting liposomal formulations have been developed to improve JQ1 delivery and efficacy in PDAC^[34]^. Furthermore, JQ1 has been reported to remodel the tumor stroma and synergize with gemcitabine^[35]^. Beyond these effects, JQ1 modulates multiple signaling nodes - such as TGF-β/Smad, PI3K–AKT, and MAPK pathways - but in some contexts may paradoxically activate oncogenic circuits^[35]^. Notably, the combination of JQ1 with CDK4/6 inhibitors such as palbociclib has not yet been explored in PDAC, and CDK4/6 inhibitors alone have not been clinically reported for this disease.

In this study, we evaluated the effects of the CDK4/6 inhibitor palbociclib (PD-0332991) and the BET inhibitor JQ1 on PDAC models both *in vitro* and *in vivo*. We show that while palbociclib monotherapy attenuates tumor cell migration, it also induces epithelial-to-mesenchymal transition (EMT) via Wnt/β-catenin activation, downstream of GSK3β phosphorylation at Ser9. This phosphorylation event is mediated by TGF-β/Smad rather than PI3K–AKT signaling. Importantly, JQ1, identified through drug library screening in this study, exhibited synergistic effects together with palbociclib in suppressing PDAC cell migration and EMT *in vivo* and *in vitro*. Mechanistically, we linked GSK3β phosphorylation status to Wnt/β-catenin activity and proposed that combined CDK4/6 and BET inhibition represents a novel therapeutic strategy for PDAC.

## METHODS

### Patient and tissue collection

Pancreatic adenocarcinoma tissues were collected from patients who underwent surgical resection between 2015 and 2019 at the First Affiliated Hospital of Dalian Medical University (*n* = 79). Each sample was independently confirmed by at least two senior pathologists. Tumor staging was determined according to the 8th edition of the tumor size, node, and metastasis (TNM) classification system of the American Joint Committee on Cancer. This study was approved by the Ethics Committee of the First Affiliated Hospital of Dalian Medical University (No. PJ-KS-KY-2024-304), and written informed consent was obtained from all participants. Patient follow-up concluded in January 2021. For construction of the tissue microarray (TMA), one representative 2.0-mm core from each case was selected by the Biobank of the First Affiliated Hospital of Dalian Medical University and arrayed onto recipient slides. Corresponding clinical data were recorded in a prospective database.

### Cancer cell lines and culture

Mouse pancreatic cancer cell lines were kindly supplied by Prof. Dr. Jens Siveke (German Cancer Research Center, GCRC). The procedures for establishing these lines have been described previously^[32,36]^. Briefly, Kras^+^/LSL-G12D, Trp53^+^/LSL-R172H, and Ptf1a^+^/Cre (KPC) mice were euthanized by cervical dislocation. Pancreatic tissues were minced and incubated in high-glucose Dulbecco’s Modified Eagle Medium (DMEM; Gibco) supplemented with 10% fetal bovine serum (FBS; Thermo Fisher, USA), 1% non-essential amino acids (Life Technologies, USA), and penicillin (50 U/mL)/streptomycin (50 µg/mL) at 37 °C. After 3-4 days, outgrowth of adherent cells was observed. Once cultures reached 50%-70% confluence, they were passaged into fresh medium. Following 5-6 passages, cells were aliquoted, named, and either used immediately for experiments or cryopreserved in liquid nitrogen for future use.

The 60400 and 70301 lines were maintained in DMEM (Solarbio, Beijing, China) supplemented with 10% FBS, 1% non-essential amino acids, and penicillin (50 U/mL)/streptomycin (50 µg/mL) at 37 °C in a humidified atmosphere with 5% CO_2_.

Human glioblastoma (U251) and lung carcinoma (A549) cell lines were generously donated by Dr. Zhikun Lin (Dalian Institute of Chemical Physics, Chinese Academy of Sciences, China). Both cell lines were cultured in high-glucose DMEM (Solarbio, Beijing, China) containing 10% FBS, 1% sodium pyruvate, 1% L-glutamine (Solarbio, Beijing, China), and penicillin (50 U/mL)/streptomycin (50 µg/mL) at 37 °C under 5% CO_2_.

### Bioinformatics analysis

Gene expression datasets (GSE28735, GSE62165, and GSE62452) were obtained from the Gene Expression Omnibus (GEO). Differentially expressed genes (DEGs) were identified using the Limma package^[37]^ in R version 4.1.1 (R Foundation for Statistical Computing, Vienna, Austria). Results were visualized as boxplots generated with ggplot2 in R. Probes without corresponding gene symbols were excluded from further analysis. Statistical significance was determined using Student’s *t*-test, with *P* values < 0.05 and |fold changes| ≥ 1 considered significant. Survival analysis was conducted using Kaplan–Meier curves and the log-rank test, based on The Cancer Genome Atlas (TCGA)-pancreatic adenocarcinoma (PAAD) data.

### Immunohistochemistry

Immunohistochemistry (IHC) was performed on formalin-fixed, paraffin-embedded mouse and human tissue sections as described previously^[32]^. Briefly, sections were incubated with the indicated primary antibodies [Table 1] for 1 h at room temperature, followed by horseradish peroxidase (HRP)-conjugated secondary antibody (KIHC-1; Proteintech, Wuhan, China) for 20 min at room temperature. Isotype IgG served as the control. All slides were independently reviewed and scored by two pathologists blinded to the clinicopathological information. Staining intensity in 10 randomly selected high-power fields (400×) was graded as: 0 (negative), 1 (weak), 2 (moderate), or 3 (strong). The percentage of positive cells in each field was scored as: 0 (< 5%), 1 (5%-25%), 2 (26%-50%), 3 (51%-75%), or 4 (> 75%). The final IHC score was calculated by multiplying intensity and percentage scores, yielding a total score of 0-12. All IHC experiments included both biological and technical replicates.

**Table 1:** Antibodies and inhibitors used in this study

**Antibodies/inhibitors**	**Source**	**Cat. No**
anti-N-Cadherin	Cell Signaling Technology	#13116
anti-E-Cadherin	Cell Signaling Technology	#3195
anti-Active β-Catenin	Cell Signaling Technology	#8814
anti-Phospho-GSK-3β (Ser9)	Cell Signaling Technology	#9323
anti-GSK-3β (D5C5Z)	Cell Signaling Technology	#12456
anti-Phospho-Smad3 (Ser423/425)	Cell Signaling Technology	#9520
anti-Smad3 (C67H9)	Cell Signaling Technology	#9523
anti-Phospho-Akt (Ser473)	Proteintech	28731-1-AP
anti-Akt (pan)	Cell Signaling Technology	#4691
anti-β-Actin (13E5)	Cell Signaling Technology	#4970
anti-BRD4	Cell Signaling Technology	#13440
anti-Phospho-Rb (Ser807/811)	Cell Signaling Technology	#8516
anti-Rb	Cell Signaling Technology	#9309
anti-c-Myc	Proteintech Group	10828-1-AP
anti-Ki-67	Servicebio Technology	GB111141
anti-Caspase3	Servicebio Technology	GB11532
anti-CDK4	Proteintech Group	11026-1-AP
anti-CDK6	Proteintech Group	14052-1-AP
anti-α-Tubulin	Proteintech Group	66031-1-Ig
anti-GAPDH	ProteinFind® TransGen Biotech	HC301-01
Goat anti-Rabbit IgG(H+L)	Proteinech Group	SA00001-2
Goat anti-Mouse IgG(H+L)	Proteintech Group	SA00001-1
PD-0332991 (Palbociclib)	APExBIO	B7798
JQ1	APExBIO	A1910
MK-2206 2HCl	Selleckchem	S1078
GDC-0941	APExBIO	A8210
LGK-974	APExBIO	B2307
LY364947	APExBIO	B2287
LY2109761	APExBIO	A8464
MSAB	Sigma-Aldrich	SML1726

### Cell viability assay

Cell viability was assessed using the CCK-8 Kit (ApexBio Biotechnology Corp., Hsinchu City, Taiwan, China). Briefly, cells were seeded into 96-well plates at an optimized density in 100 µL of medium containing 10% FBS. After 6 h, 100 µL of serially diluted compounds [Table 1] or dimethyl sulfoxide (vehicle) was added, and cells were cultured for 0, 24, 48, or 72 h. Drug concentration ranges were based on Selleck references (https://www.selleck.cn/). To evaluate the combined effects of PD-0332991 and JQ1 [Table 1] on PDAC cell viability, drug interactions were analyzed using Compusyn software (http://www.combosyn.com/index.html). Cell viability was determined by CCK-8 assay as described above, and the fixed-effect model was applied to calculate the combination index (CI) according to the software protocol. CI values < 1, = 1, and > 1, indicated “synergism”, “additive effects”, and “antagonism”, respectively.

### Wound healing assay

Cells were seeded into 6-well plates at a density of 1 × 10^6^ cells per well. Once a confluent monolayer formed, a linear scratch was created using a sterile pipette tip. The cells were then gently washed twice with 1× PBS buffer and incubated in serum-free medium containing the specified concentrations of drugs. Plates were maintained at 37 °C and monitored using an inverted phase contrast microscope (Zeiss, Germany). Images were captured at predefined time points, such as 0 and 24 h, using the Zeiss image analysis program. Wound closure was quantified using ImageJ software, and the results were visualized as bar charts generated with Prism^TM^ version 8.3.0 (GraphPad, USA).

### Transwell invasion assay

A total of 2 × 10^5^ cells were seeded into Transwell chambers with 8-μm pores (BIOFIL), pre-coated with Matrigel (Thermo Fisher Scientific, USA), and placed in 24-well culture plates. After 72 h of incubation, cells that had migrated to the lower surface of the membrane were fixed and stained with 0.1% crystal violet. Images of invasive cells were captured using a microscope, and cell numbers were quantified using ImageJ software. Data were presented as bar graphs generated with Prism^TM^ version 8.3.0 (GraphPad, USA).

### Labeling with 5-ethynyl-2′-deoxyuridine

Cells were labeled with 5-ethynyl-2′-deoxyuridine (EdU; APExBio, USA) following the manufacturer’s protocol. Briefly, cells were seeded into 6-well plates at a density of 1 × 10^5^ cells per well and incubated at 37 °C for 24 h, followed by treatment with inhibitors at defined concentrations for an additional 72 h. After treatment, cells were incubated with 20 μM EdU at 37 °C for 2 h, fixed in 4% formaldehyde for 15 min, and permeabilized with 0.5% (v/v) Triton X-100 at room temperature for 20 min. After three PBS washes, cells were incubated with the Click iT^TM^ reaction cocktail (Thermo Fisher, USA) in the dark for 30 min. Finally, cells were stained with Hoechst for 30 min and visualized using a confocal microscope (Leica Microsystems GmbH, Wetzlar, Germany).

### Real-time quantitative polymerase chain reaction

Total RNA was extracted using TRIzol reagent (Invitrogen, USA), and complementary DNA (cDNA) was synthesized with PrimeScript RT Reagent Kits (Takara Biotech, Japan). Quantitative polymerase chain reaction (qPCR) was performed on an ABI QuantStudio3 Real-Time PCR system (Thermo Fisher Scientific, USA) using SYBR Green reagents (Takara Biotech, Japan). Gene expression levels were normalized to GAPDH. The forward and reverse primers were as follows: CDH1-f, 5′-CAGGTCTCCTCATGGCTTTGC-3′ and CDH1-r, 5′-CTTCCGAAAAGAAGGCTGTCC-3′; CDH2-f, 5′-AGCGCAGTCTTACCGAAGG-3′ and CDH2-r, 5′-TCGCTGCTTTCATACTGAACTTT-3′; VIM-f, 5′-CGTCCACACGCACCTACAG-3′ and VIM-r, 5′-GGGGGATGAGGAATAGAGGCT-3′; SNAI2-f, 5′-TGGTCAAGAAACATTTCAACGCC-3′ and SNAI2-r, 5′-GGTGAGGATCTCTGGTTTTGGTA-3′; GAPDH-f, 5′-AGGTCGGTGTGAACGGATTTG-3′ and GAPDH-r, 5′-TGTAGACCATGTAGTTGAGGTCA-3′.

### Western blotting

Cells were lysed in RIPA buffer (Sangon Biotech, China) for 30 min, and total protein was quantified using the BCA Assay Kit (Sangon Biotech, China). The RIPA buffer contained 10 mM Tris-HCl (pH 8.0), 150 mM NaCl, 1 mM EDTA, 0.5 mM EGTA, 1% (v/v) Triton X-100, 0.1% (m/v) SDS, protease inhibitor cocktail, and phosphatase inhibitor cocktail. Lysates were separated by 10% reducing SDS-PAGE, and protein bands were transferred onto nitrocellulose membranes. Membranes were incubated with the appropriate primary antibodies [Table 1] at optimal dilutions overnight at 4 °C. After washing with TBST, membranes were incubated with HRP-conjugated goat anti-rabbit or goat anti-mouse secondary antibodies. Immunoblots were imaged using a Chemiluminescent Imaging System (Thermo Fisher Scientific, USA). All experiments included biological and technical replicates.

### Xenograft evaluation

Six-week-old BALB/c nude mice were randomly divided into four groups to receive PD-0332991 monotherapy, JQ1 monotherapy, combination therapy, or vehicle control (sodium carboxymethylcellulose, CMC). PDAC cells (5 × 10^5^) were suspended in 100 µL of Geltrex® Matrigel (Thermo Fisher Scientific, USA) diluted 4:1 (v/v) in PBS, and injected subcutaneously into the backs of mice. Tumor size was calculated as *l* × *w*^2^, where *l* and *w* represent the longest and shortest diameters, respectively. Drug administration began when tumors reached ~0.5 cm^3^, with doses based on previous studies^[38,39]^. Mice were weighed, and tumor volumes were recorded every three days. At the study endpoint, mice were euthanized and tumors harvested for further analysis. All animal procedures were approved by the Animal Ethics Committee of Dalian Medical University (No. AEE21032).

### Statistical analysis

Statistical analysis and figure creation were conducted using R version 4.1.1 (R Foundation for Statistical Computing, Vienna, Austria) and GraphPad Prism version 8 (GraphPad Software Inc., USA). Continuous variables were transformed into categorical variables using appropriate cut-off values and analyzed with the chi-square (χ^2^) test or Fisher’s exact test. Paired *t*-tests were employed for data comparisons. Overall survival (OS) was assessed with Kaplan–Meier curves and compared using log-rank tests. Model performance was evaluated with the concordance index (C-index) and the area under the curve (AUC). Data are presented as mean ± SEM (*N* = 3 or as specified). Statistical significance was defined as: ^*^P < 0.05, ^**^P < 0.01, ^***^P < 0.001, ^****^P < 0.0001; “ns” indicates no significant difference as determined by Student’s *t*-test.

## RESULTS

### CDK4 and CDK6 are upregulated in PDAC and associated with poor OS

**Figure 1:**
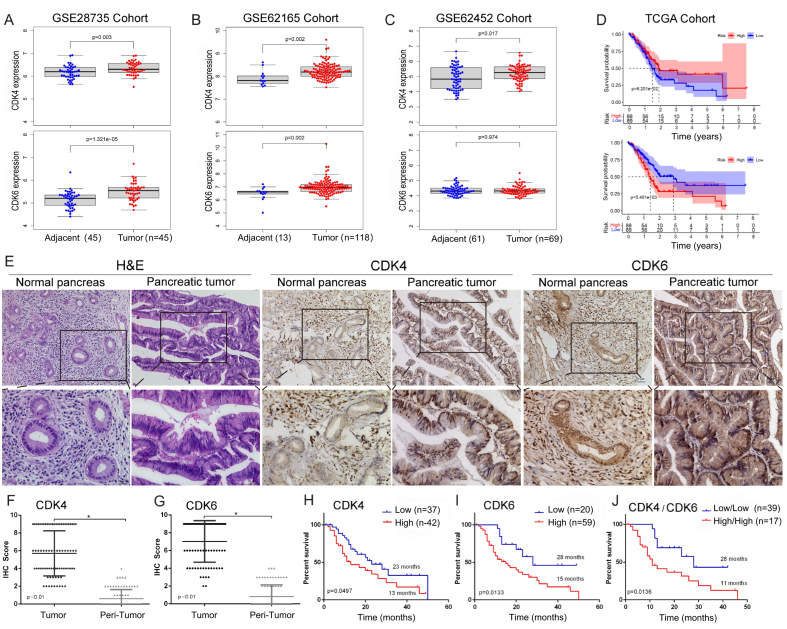
mRNA and protein expression of CDK4/6 and their correlation with OS. Differential expression patterns of CDK4 and CDK6 in (A) GSE28735, (B) GSE62165, and (C) GSE62452 cohorts; (D) Association between CDK4/6 expression and OS in the TCGA cohort; (E) Representative immunohistochemical staining of CDK4 and CDK6 in tumor and adjacent non-tumor tissues. Scales bars, 100 μm; (F and G) Quantification of CDK4 and CDK6 in 79 paired tumor and adjacent non-tumor tissues; (H and I) Association of CDK4 and CDK6 protein expression with OS in our cohort; (J) Kaplan–Meier survival analysis comparing patients with high CDK4 and CDK6 expression (CDK4^high^/6^high^) *vs.* low expression (CDK4^low^/6^low^). Data are presented as mean ± SEM from three experiments. ^*^P < 0.05. CDK4/6: Cyclin-dependent kinases 4 and 6; OS: overall survival; TCGA: The Cancer Genome Atlas; SEM: standard error of the mean.

We first analyzed the differential expression of CDK4 and CDK6 at the mRNA level between pancreatic cancer and non-cancerous tissues using the GSE28735 (*N* = 90), GSE62165 (*N* = 131), and GSE62452 (*N* = 130) cohorts. Except for CDK6 in the GSE62452 cohort, both CDK4 and CDK6 were highly expressed in PDAC tissues [Figure 1A-C]. Kaplan–Meier analysis based on the TCGA database indicated that elevated CDK6 expression was significantly associated with worse prognosis. Although CDK4 expression showed a negative trend with OS, this association did not reach statistical significance (*P* = 0.062 for CDK4; *P* = 0.0054 for CDK6) [Figure 1D].

Next, we assessed CDK4 and CDK6 protein expression levels by IHC in our cohort. A total of 79 paired, pathologically confirmed PDAC and adjacent non-cancerous tissues were collected from two centers using TMAs [Figure 1E]. Both CDK4 and CDK6 were significantly upregulated in PDAC tissues compared to non-cancerous controls [Figure 1F and G]. Kaplan–Meier analysis further demonstrated that higher CDK4 and CDK6 expression levels were significantly associated with shorter OS (*P* = 0.0497 for CDK4; *P* = 0.0133 for CDK6) [Figure 1H and I]. Notably, patients with high expression of both CDK4 and CDK6 (CDK4^high^/6^high^) had poorer prognosis than those with low expression (CDK4^low^/6^low^) [Figure 1J]. Correlation analysis with clinicopathological variables revealed that CDK4 expression was significantly associated with lymphatic metastasis, advanced N stage, and receipt of chemotherapy [Table 2].

**Table 2:** Relationship between clinicopathologic parameters and CDK4, CDK6 expression in pancreatic cancer (*N* = 79)

**Characteristics**	***N***	**CDK4**			**CDK6**		
		**High**	**Low**	***P*-value**	**High**	**Low**	***P*-value**
**Age**				0.397			0.970
≥ 60	59	33	26		44	15	
< 60	20	9	11		15	5	
**Gender**				0.780			0.940
Male	54	24	30		33	11	
Female	35	18	17		26	9	
**Tumor size**				0.888			0.710
≤ 3	25	13	12		18	7	
> 3	54	29	25		41	13	
**Diabetes**				0.550			0.320
Yes	21	10	11		14	7	
No	58	32	26		45	13	
**CEA**				0.625			0.830
≤ 5 μg/L	49	25	24		37	12	
> 5 μg/L	30	17	13		22	8	
**CA19-9**				0.580			0.708
≤ 27 kU/L	10	4	6		7	3	
> 27 kU/L	69	38	31		52	17	
**Location**				0.650			0.100
Head	47	24	23		32	15	
Body and tail	32	18	14		27	5	
**Perineural invasion**				1.000			0.445
Yes	77	41	36		58	19	
No	2	1	1		1	1	
**Lymphatic Met.**				0.045^*^			0.160
Yes	26	18	8		22	4	
No	53	24	29		37	16	
**Tumor differentiation**				1.000			0.351
High	17	9	8		15	2	
Moderate	53	28	25		37	16	
Poor	9	5	4		7	2	
**Peri-invasion**				0.530			0.460
Yes	54	30	24		39	15	
No	25	12	13		20	5	
**Microvascular invasion**				1.000			1.000
Yes	7	4	3		5	2	
No	72	38	34		54	18	
**Tumor embolus**				0.169			0.640
Yes	23	15	8		18	5	
No	56	27	29		41	15	
Chemotherapy				0.004^*^			0.048^*^
Yes	22	6	16		13	9	
No	57	36	21		46	11	
**T stage**				0.885			0.910
T1	9	5	4		7	2	
T2	49	25	24		37	12	
T3	21	12	9		15	6	
T4	0	0	0		0	0	
**N stage**				0.035^*^			0.347
N0	54	24	30		38	16	
N1	21	14	7		18	3	
N2	4	4	0		3	1	
**M stage**				0.957			0.370
M0	66	35	31		48	18	
M1	13	7	6		11	2	
**TNM stage**				0.398			0.710
I	34	15	19		24	10	
II	29	17	12		22	7	
III-IV	16	10	6		13	3	

^*^P < 0.05 indicates statistical significance. CDK4/6: Cyclin-dependent kinases 4 and 6; TNM: tumor size, node, and metastasis.

### Stratification by CDK4/6 as a prognostic predictor in PDAC

**Figure 2:**
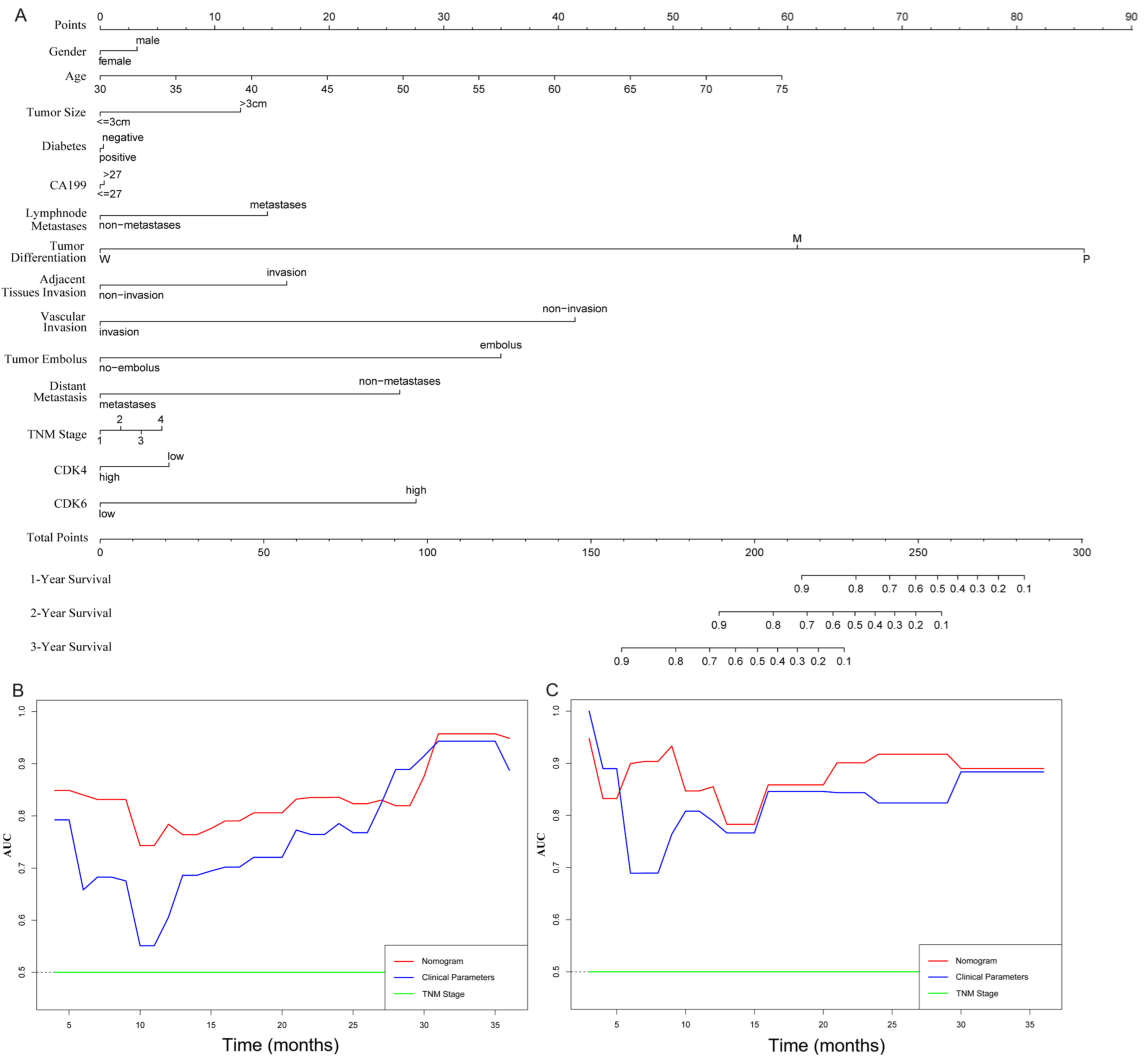
Establishment and evaluation of a CDK4/6-based nomogram in PDAC cohorts. (A) Nomogram parameters, including gender, age, tumor size, diabetes, CA199 level, lymphatic metastasis, tumor differentiation, perineural invasion, tumor embolus, distant metastasis, TNM stage, and CDK4/6 expression, were used to predict 1-, 2- and 3-year survival in PDAC patients; (B and C) Time-dependent ROC curves comparing the nomogram, clinical parameters, and TNM stage in the training and validation cohorts. CDK4/6: Cyclin-dependent kinases 4 and 6; PDAC: pancreatic ductal adenocarcinoma; TNM: tumor size, node, and metastasis; ROC: receiver operating characteristic.

We next evaluated whether combining CDK4/6 expression with clinicopathological variables could improve prognostic accuracy. A cohort of 46 PDAC patients was used as the training set to construct a composite prognostic nomogram incorporating gender, age, tumor size, diabetes, CA199 level, lymphatic metastasis, tumor differentiation, perineural invasion, tumor embolus, distant metastasis, TNM stage, and CDK4/6 expression to predict 1-, 2-, and 3-year survival [Figure 2A]. The remaining 23 PDAC patients served as the validation set. Prognostic performance of the nomogram, TNM stage, and clinical parameters was compared using time-dependent receiver operating characteristic (ROC) curves (AUC) and C-index. Our analysis demonstrated that the nomogram provided superior prognostic accuracy compared with the clinical parameters [Figure 2B] and TNM stage [Figure 2C]. The C-index for OS prediction was significantly higher for the nomogram than for either TNM Stage or clinical parameters (*P* < 0.05, Table 3). These results suggest that incorporating CDK4/6 expression with clinical variables improves prognostic prediction in PDAC.

**Table 3:** C-index of predictive accuracy for different models

**Model**	**Training cohort**	**Test cohort**
Nomogram	0.778	0.861
Clinical parameters	0.751	0.693
TNM stage	0.553	0.563

TNM: Tumor size, node, and metastasis.

### CDK4/6 inhibitor PD-0332991 slightly suppresses tumor growth but promotes migration and invasion

**Figure 3:**
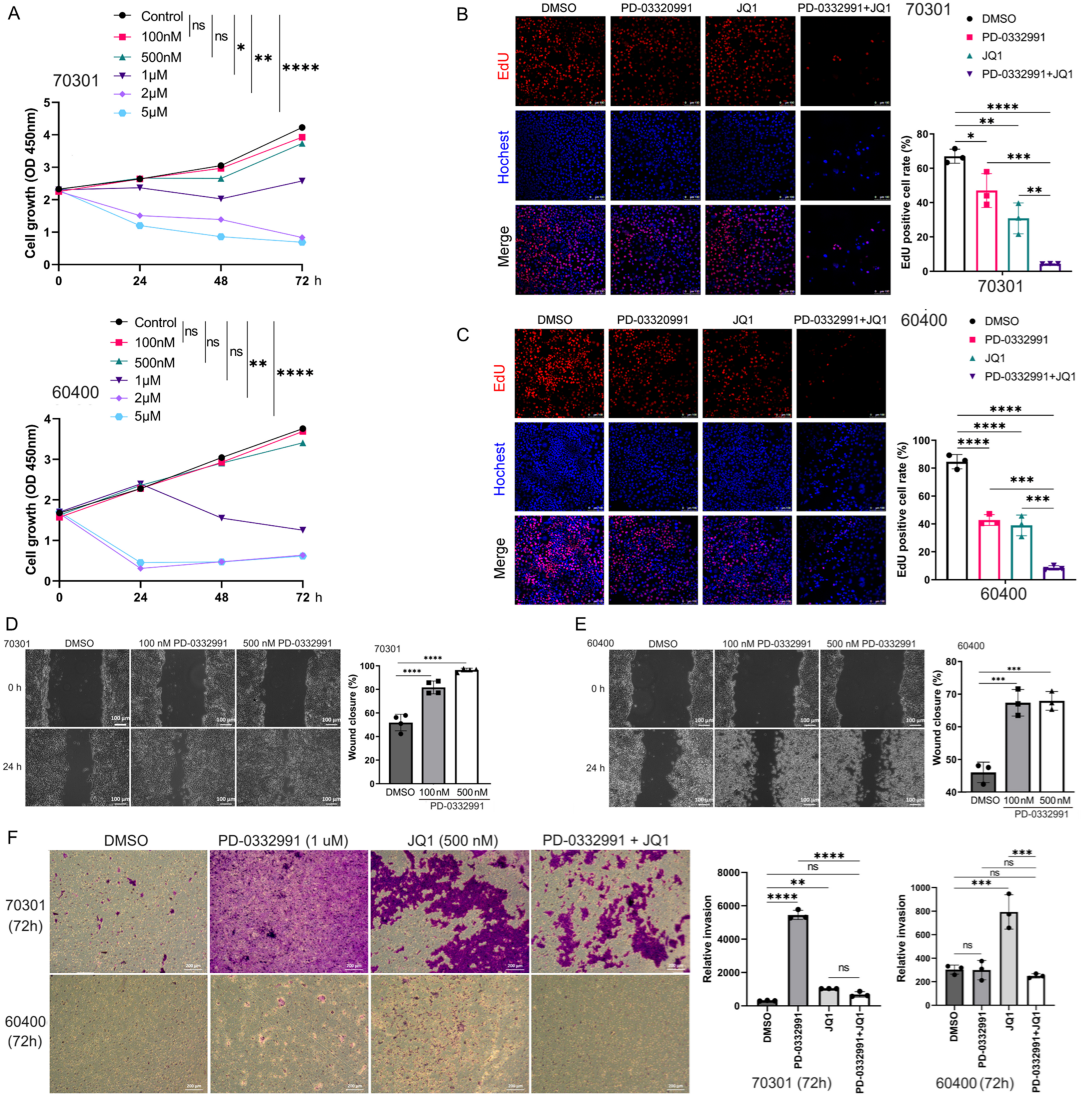
Effects of PD-0332991 on two primary PDAC cell lines *in vitro*. (A) Time- and dose-dependent effects of PD-0332991 on 70301 (upper) and 60400 (lower) cells; (B and C) EdU assay showing cell viability after treatment with PD-0332991 (1 μM), with or without JQ1 (500 nM). Scales bars,100 μm; (D) Wound healing assays showing enhanced migration following 100 and 500 nM PD-0332991 treatment for 24 h. Scales bars, 100 μm; (E) Quantification of wound healing assays for 70301 and 60400 cells; (F) Transwell invasion assays demonstrating effects of PD-0332991 and JQ1. Scales bars, 200 μm. Data are mean ± SEM from three independent experiments or three randomly selected fields per experiment. ^*^P < 0.05, ^**^P < 0.01, ^***^P < 0.001, ^****^P < 0.0001; ns: not significant. PDAC: Pancreatic ductal adenocarcinoma; EdU: 5-ethynyl-2′-deoxyuridine; SEM: standard error of the mean.

Two primary PDAC cell lines (70301 and 60400) derived from *KPC* mice were treated with PD-0332991 at predefined concentrations for 24, 48, and 72 h. PD-0332991 at 1-5 μM, inhibited 70301 cell growth in a time-dependent manner. Treatment with 2 μM PD-0332991 for 24 h significantly suppressed the proliferation of 60400 cells [Figure 3A]. EdU assays further confirmed that PD-0332991 monotherapy inhibited cell viability in both cell lines [Figure 3B and C]. However, wound healing assays revealed that PD-0332991 (100 and 500 nM) promoted cell migration, more significantly in 70301 cells than in 60400 cells [Figure 3D and E]. Similarly, transwell assays indicated that PD-0332991 enhanced invasive capacity in both cell lines [Figure 3F]. The pro-migratory and pro-invasive effects of PD-0332991 are likely mediated by compensatory signaling pathways activated via EMT or cell cycle arrest, rather than by proliferation inhibition alone. Collectively, these findings suggest that PD-0332991 monotherapy slightly inhibits cell growth while concurrently promoting PDAC cell migration and invasion.

### PD-0332991 induces EMT in PDAC

**Figure 4:**
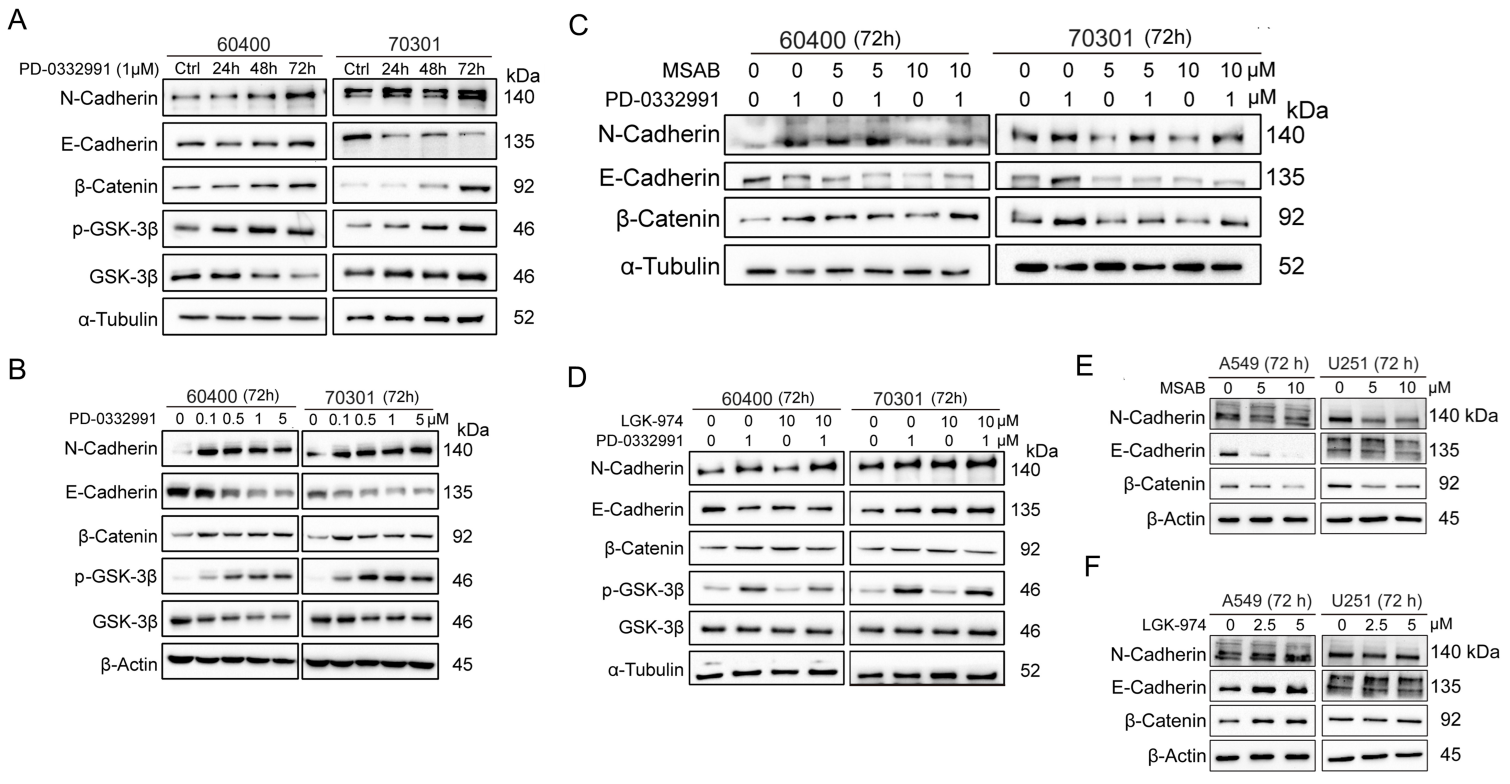
PD-0332991 promotes EMT through activation of the canonical Wnt/β-catenin pathway in PDAC cells. PD-0332991 induced EMT in a time- and dose-dependent manner by activating canonical Wnt/β-catenin signaling, with the strongest effect on N-cadherin. (A) Primary PDAC cell lines 60400 and 70301 cells treated with 1 μM PD-0332991 for 24, 48, and 72 h; (B) Dose-dependent effects of PD-0332991 (0.1-5 μM for 72 h) on 60400 and 70301 cells; (C) Direct inhibition of β-catenin with the small-molecule inhibitor MSAB suppressed PD-0332991-induced EMT and attenuated Wnt/β-catenin pathway activation; (D) Treatment with the PORCN inhibitor LGK974 altered EMT progression and Wnt/β-catenin signaling in 60400 and 70301 cells; (E and F) Inhibition of Wnt/β-catenin signaling, either by targeting PORCN or directly inhibiting β-catenin with MASB, also suppressed EMT in non-small lung cell carcinoma (A549) and glioma (U251) cells. EMT: Epithelial-to-mesenchymal transition; PDAC: pancreatic ductal adenocarcinoma.

EMT is closely associated with enhanced migration, invasion, and metastasis. We investigated the mechanism by which PD-0332991 contributes to EMT in PDAC. Time-course experiments demonstrated that 1 μM PD-0332991 increased N-cadherin and decreased E-cadherin expression in 60400 and 70301 cells [Figure 4A]. Additionally, active (non-phosphorylated) β-catenin levels were significantly upregulated in a dose-dependent manner [Figure 4B].

### PD-0332991-induced EMT is mediated primarily via Wnt/β-catenin signaling

To determine whether EMT induction involved the Wnt/β-catenin pathway, we used two pathway inhibitors: MSAB, a direct β-catenin inhibitor, and LGK974, a PORCN inhibitor, in combination with PD-0332991 in 60400 and 70301 cells. Western blotting showed that MSAB significantly reduced N-cadherin, whereas E-cadherin was decreased but not rescued [Figure 4C]. β-catenin levels were unaffected by LGK974 in both cell lines [Figure 4D]. Interestingly, MSAB did not reduce β-catenin in 70301 cells and even increased it in 60400 cells [Figure 4C]. These results suggest that suppression of N-cadherin, rather than E-cadherin, is associated with EMT inhibition through Wnt/β-catenin pathway inhibition. To validate these findings, U251 (glioma) and A549 (non-small cell lung cancer) cells were also treated with MSAB or LGK974 alongside PD-0332991 [Figure 4E and F]. Despite Wnt/β-catenin pathway inhibition, both N-cadherin and E-cadherin decreased [Figure 4E and F], indicating that PD-0332991 induces EMT predominantly via Wnt/β-catenin signaling.

### Activation of β-catenin via p-GSK3β after PD-0332991 treatment is independent of the PI3K/AKT pathway

**Figure 5:**
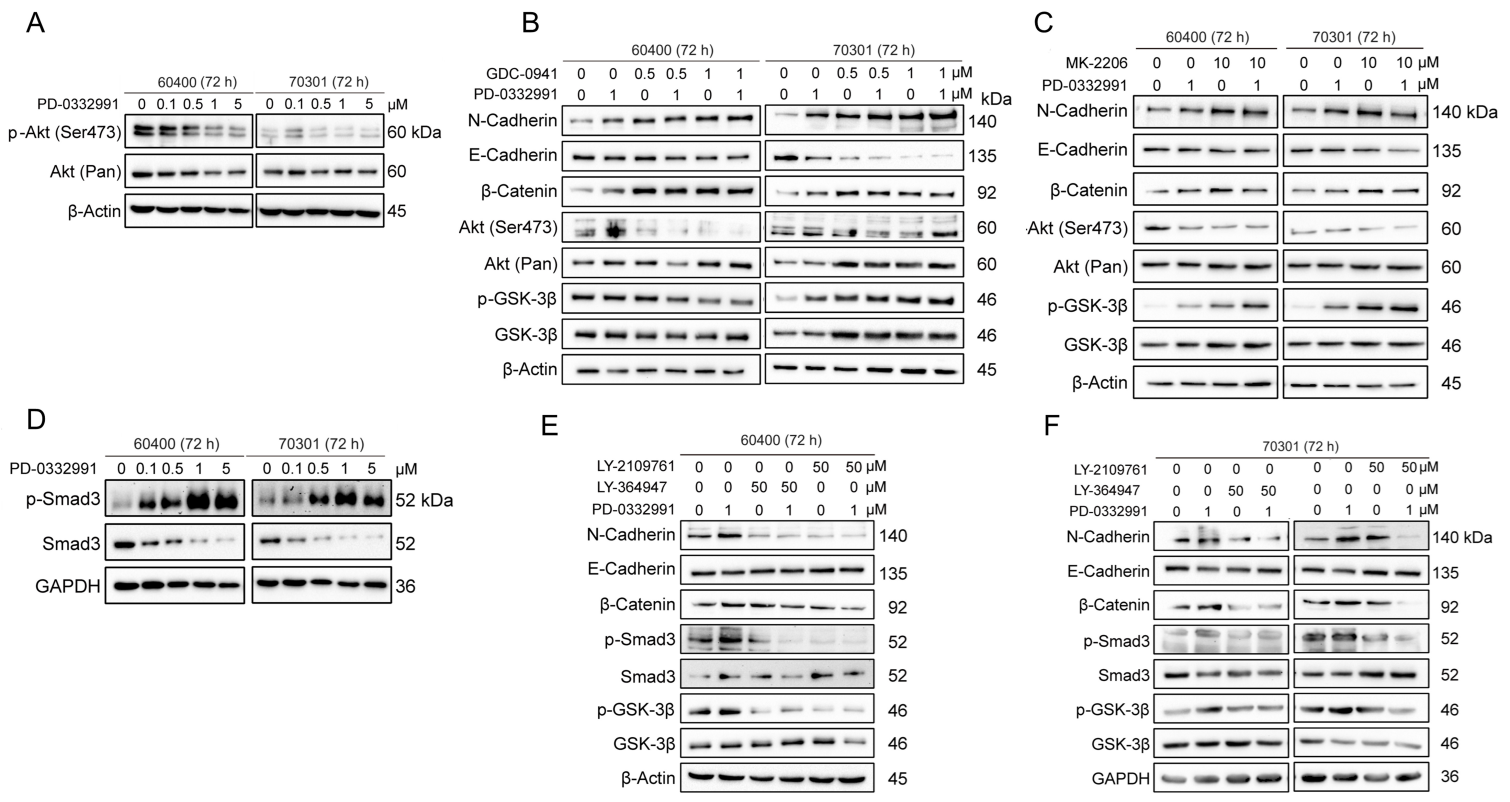
TGFβ/Smad, but not the PI3K-AKT pathway, mediates activation of the Wnt/β-catenin pathway through GSK3β Ser9 phosphorylation in 60400 and 70301 cells treated with the CDK4/6 inhibitor PD-0332991 for 72 h. (A) PD-0332991 treatment did not increase p-AKT (Ser473) levels in pancreatic cancer cells; (B) Inhibition of the PI3K-AKT pathway at the PI3Kα/δ node with GDC0941 failed to reverse EMT or suppress Wnt/β-catenin signaling, but instead enhanced EMT; (C) Inhibition of the PI3K-AKT pathway at the AKT node with MK2206 also failed to reverse EMT or suppress Wnt/β-catenin signaling, while also promoting EMT; (D) PD-0332991 activated the TGFβ/Smad pathway by upregulating pSmad3 in pancreatic cancer cells; (E and F) Inhibition of the TGFβ/Smad pathway with the small molecule inhibitors LY364947 or LY2109761 suppressed EMT and Wnt/β-catenin signaling. CDK4/6: Cyclin-dependent kinases 4 and 6; EMT: epithelial-to-mesenchymal transition.

Phosphorylation at Ser9 is known to inactivate GSK3β, thereby preventing the proteasomal degradation of β-catenin. This canonical phosphorylation event is typically regulated by the PI3K/AKT pathway. To explore the molecular mechanisms by which PD-0332991 activates the Wnt/β-catenin pathway through GSK3β, we examined the potential involvement of the PI3K/AKT signaling axis, which is known to cross-talk with Wnt/β-catenin and plays a crucial role in tumor growth, invasion, and metastasis. Our results revealed that PD-0332991 treatment (≥ 1 μM) did not significantly affect p-AKT (Ser473) levels, suggesting that AKT activation is not involved in CDK4/6 inhibition-mediated effects [Figure 5A]. Furthermore, treatment with the PI3K inhibitor GDC0941 [Figure 5B], the AKT inhibitor MK2206 [Figure 5C], or their combination failed to suppress GSK3β expression or active β-catenin levels**,** and phosphorylation of GSK3β remained largely unchanged. These findings indicate that β-catenin activation after PD-0332991 treatment occurs independently of the PI3K/AKT pathway.

### TGFβ/Smad signaling regulates Wnt/β-catenin activation via GSK3β phosphorylation after PD-0332991 treatment

To investigate how the CDK4/6 inhibitor PD-0332991 activates Wnt/β-catenin signaling by promoting β-catenin dephosphorylation through GSK3β inactivation, we examined the role of the TGFβ/Smad pathway, a key signaling axis associated with EMT. Our results demonstrated that phosphorylated Smad3 was significantly upregulated after PD-0332991 treatment, indicating activation of the TGFβ/Smad pathway [Figure 5D]. Co-treatment with PD-0332991 and the TGFβ receptor I/II inhibitors LY364947 and LY2109761 reduced levels of phosphorylated Smad3 and GSK3β, which in turn suppressed Wnt/β-catenin signaling and partially reversed EMT in 60400 and 70301 cells [Figure 5E and F]. Previous studies have similarly shown that TGFβ/Smad3 signaling upregulates the expression of several Wnt ligands (2b, 4, 5a, 9a, and 11) and enhances β-catenin stability^[40]^. Collectively, these findings suggest that TGFβ/Smad signaling contributes to Wnt/β-catenin activation by inducing Ser9 phosphorylation of GSK3β in response to PD-0332991 treatment.

### BET inhibitor JQ1 inhibits tumor growth but promotes tumor migration, similar to PD-0332991

**Figure 6:**
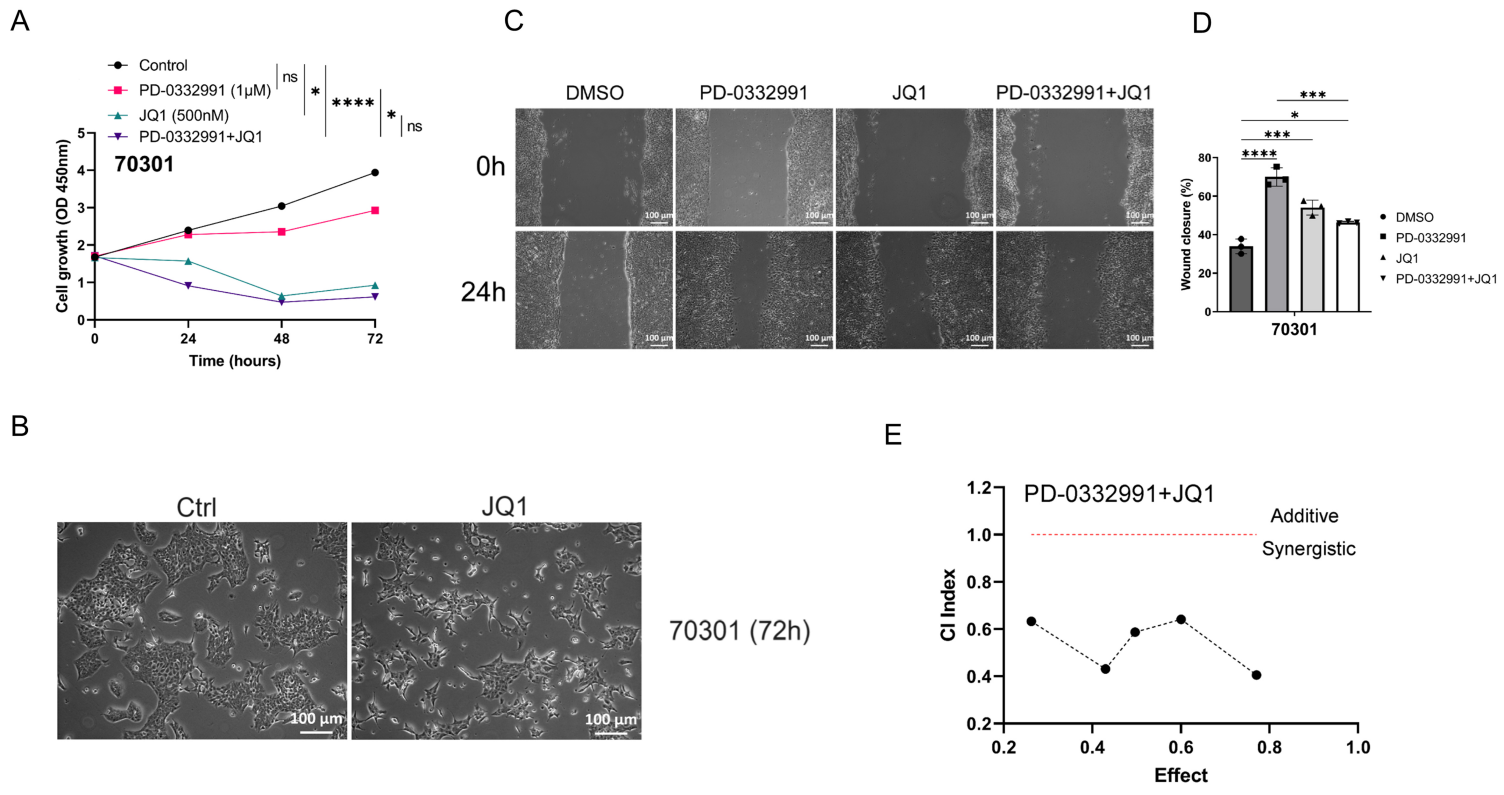
Combined JQ1 and PD-0332991 synergistically suppress tumor growth, migration, and invasion. (A) The combination of JQ1 and PD-0332991 significantly suppressed tumor growth in 70301 cells; (B) Morphological changes in 70301 cells before and after JQ1 monotherapy. Scales bars, 100 μm; (C and D) Effects of PD-0332991, JQ1, and their combination on the migration of 70301 cells. Scales bars, 100 μm; (E) CI analysis of JQ1 and PD-0339221. Cell viability was measured using the CCK-8 assay after 72 h of treatment in 70301 cells. All data are presented as mean ± SEM from at least three independent experiments, with three randomly selected fields per experiment. ^*^P < 0.05; ^***^P < 0.001; ^****^P < 0.0001; ns: not significant. CI: Combination index; SEM: standard error of the mean.

**Figure 7:**
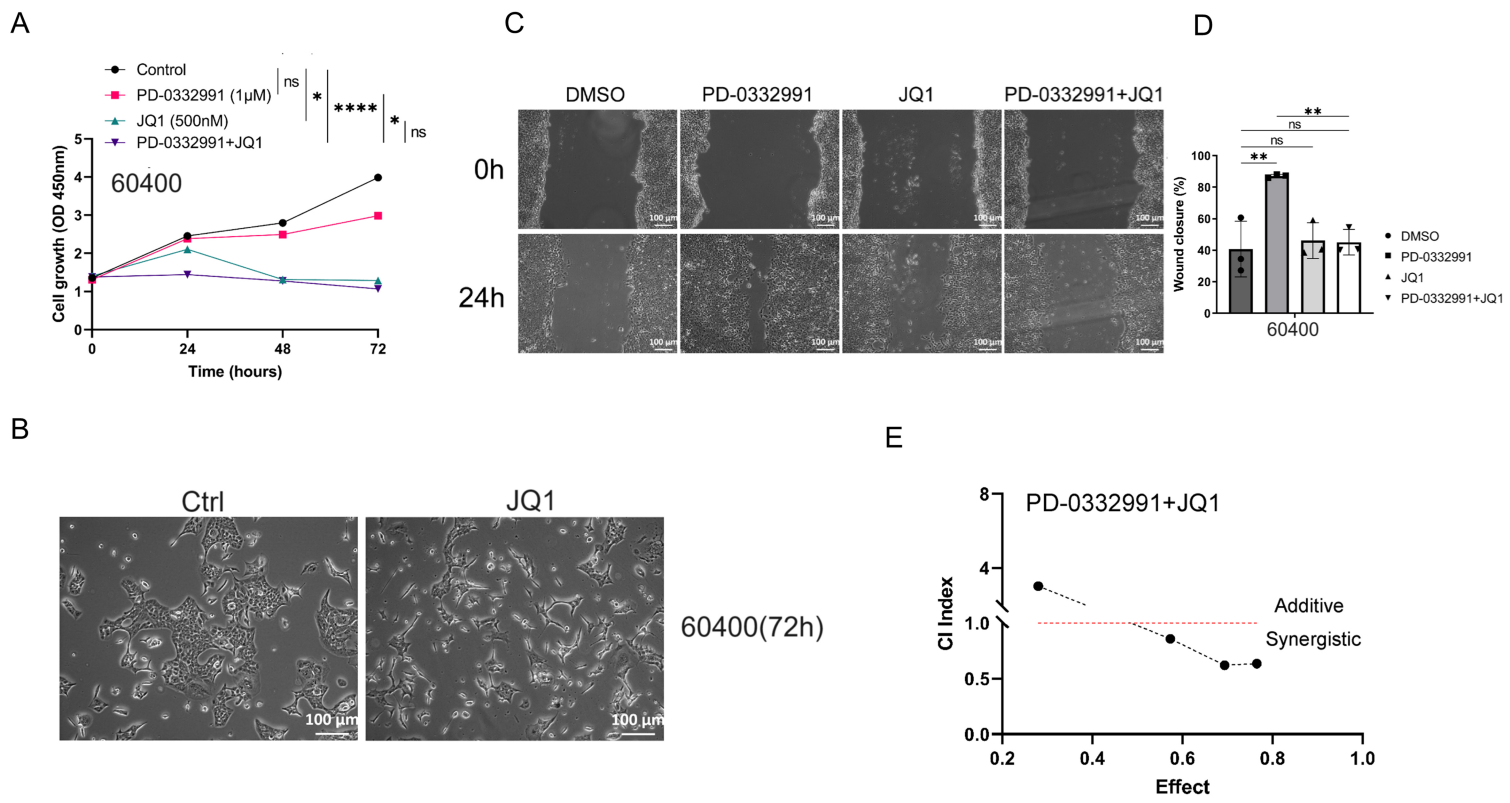
(A) Combined JQ1 and PD-0332991 significantly suppressed tumor growth in 60400 cells; (B) Changes in cell morphology before and after JQ1 monotherapy in 60400 cells. Scales bars, 100 μM; (C and D) Effects of PD-0332991, JQ1, and their combination on the migration of 60400 cells. Scales bars, 100 μM; (E) Combined treatment with JQ1 and PD-0332991 significantly suppressed tumor growth in 60400 cells. Data are presented as mean ± SEM from at least three independent experiments, with three randomly selected fields per experiment. ^*^P < 0.05; ^**^P < 0.01; ^****^P < 0.0001; ns: not significant, Student’s *t*-test. SEM: Standard error of the mean.

In a similar experimental setup, two primary cell lines derived from *KPC* mice were separately treated with JQ1, PD-0332991, or their combination at defined concentrations for 24, 48, and 72 h. Treatment with 0.5 μM JQ1 inhibited the growth of 70301 cells [Figure 6A] and 60400 cells [Figure 7A]. Notably, exposure to 0.5 μM JQ1 for 72 h, as well as combined treatment with 1 μM PD-0332991 and 0.5 μM JQ1 for 72 h, significantly suppressed the proliferation of both 70301 [Figure 6A] and 60400 cells [Figure 7A]. EdU assay results further showed that PD-0332991 monotherapy slightly reduced the viability of 70301 [Figure 3B] and 60400 cells [Figure 3C]. Morphological changes in cells following JQ1 monotherapy are shown in Figures 6B and 7B. Taken together, these findings suggest that 0.5 μM JQ1 significantly suppresses cell growth, and that its combination with PD-0332991 leads to near-complete growth inhibition. However, wound healing assays revealed that treatment with 1 μM PD-0332991, 0.5 μM JQ1, or their combination markedly promoted the migration of 70301 cells [Figure 6C and D], whereas PD-0332991 alone had limited effects on migration in 60400 cells [Figure 7C and D]. Consistently, transwell assays suggested that PD-0332991 may enhance the invasive potential of both 70301 and 60400 cells [Figure 3F]. Collectively, these results indicate that the BET inhibitor JQ1, similar to PD-0332991, inhibits PDAC cell growth but paradoxically promotes cell migration and may suppress invasion.

### BET inhibitor JQ1 combined with PD-0332991 synergically suppresses tumor growth, migration, and invasion

**Figure 8:**
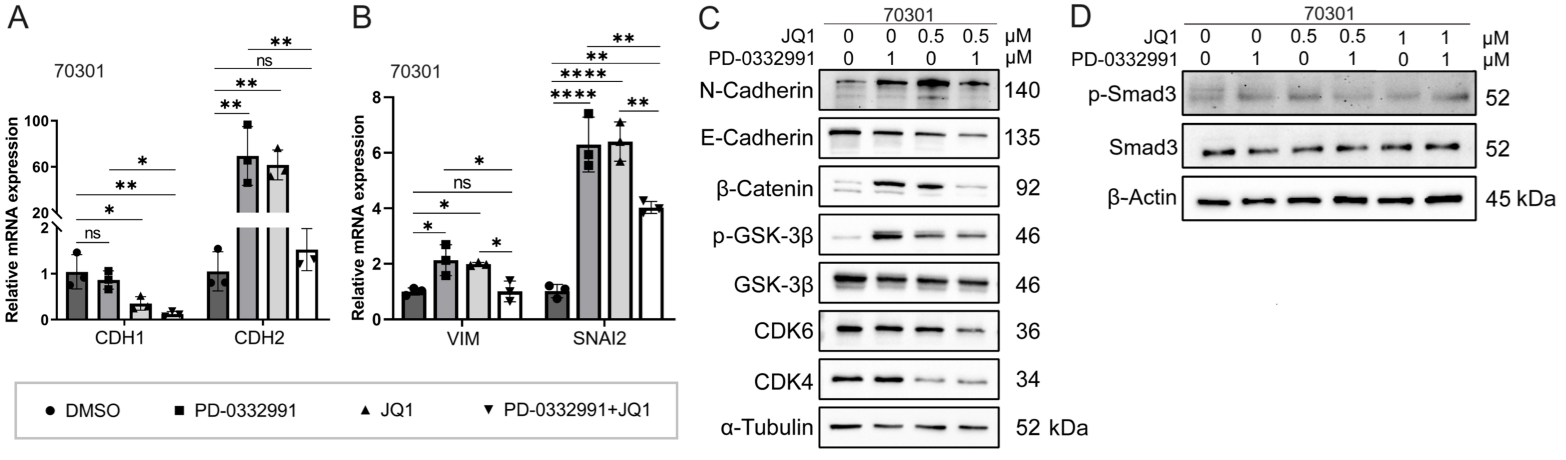
Mechanism effects of combined JQ1 and PD-0332991 treatment on EMT in pancreatic 70301 cells. (A) qPCR analysis of EMT-related gene expression in 70301 cells treated with PD-0332991, with or without JQ1; (B) qPCR analysis of invasion-associated gene expression in 70301 cells treated with PD-0332991, with or without JQ1; (C) Western blot analysis of EMT markers and Wnt/β-catenin signaling components following treatment with PD-0332991, JQ1, or their combination in 70301 cells; (D) Western blot analysis of TGFβ/Smad pathway inhibition by JQ1 in 70301 cells. Data are presented as mean ± SEM from three independent experiments. ^*^P < 0.05, ^**^P < 0.01, ^****^P < 0.0001; ns: not significant. EMT: Epithelial-to-mesenchymal transition; qPCR: quantitative polymerase chain reaction; SEM: standard error of the mean.

**Figure 9:**
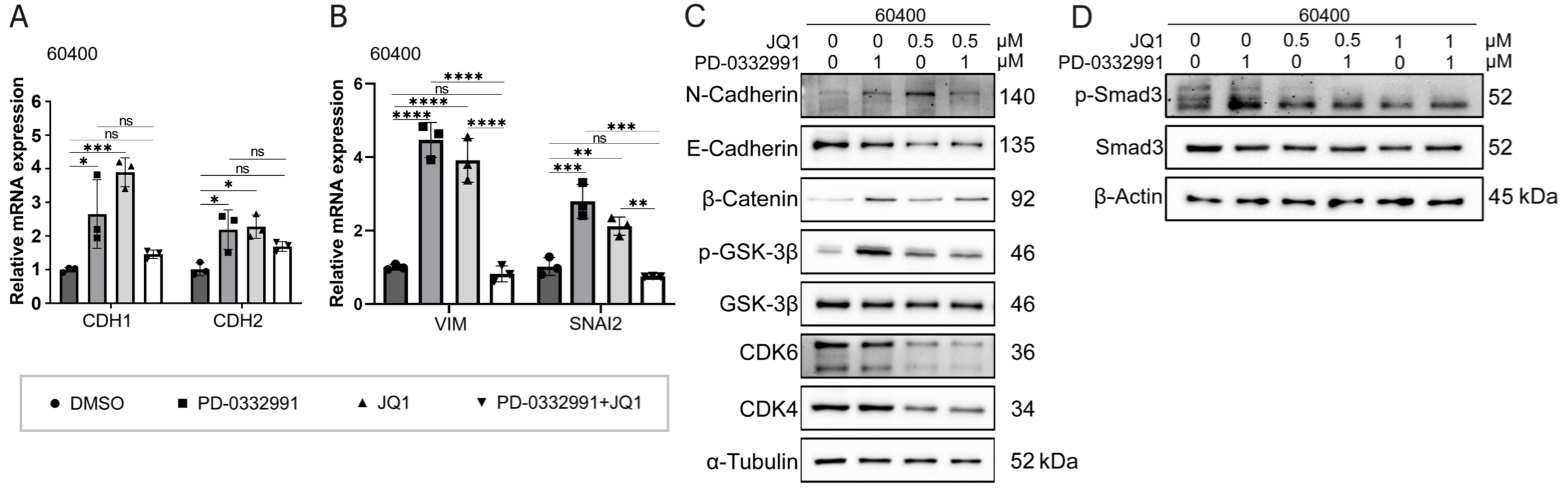
Mechanism effects of combined JQ1 and PD-0332991 treatment on EMT in pancreatic 60400 cells. (A) qPCR analysis of EMT-related gene expression in 60400 cells treated with PD-0332991, with or without JQ1; (B) qPCR analysis of invasion-associated gene expression in 60400 cells treated with PD-0332991, with or without JQ1; (C) Western blot analysis of EMT markers and Wnt/β-catenin signaling components following treatment with PD-0332991, JQ1, or their combination in 60400 cells; (D) Western blot analysis of TGFβ/Smad pathway inhibition by JQ1 in 60400 cells. Data are presented as mean ± SEM from three independent experiments. ^*^P < 0.05, ^**^P < 0.01, ^***^P < 0.001, ^****^P < 0.0001; ns: not significant. EMT: Epithelial-to-mesenchymal transition; qPCR: quantitative polymerase chain reaction; SEM: standard error of the mean.

Through drug library screening, we identified the BET inhibitor JQ1 as capable of counteracting the adverse effects of PD-0332991 while exerting a strong synergistic effect. The CCK-8 assay showed that JQ1 enhanced the inhibitory activity of PD-0332991 [Figures 6A and 7A], and CI analysis using CompuSyn confirmed a synergistic effect between the two inhibitors *in vitro* [Figures 6E and 7E]. This finding was further supported by EdU assay results [Figure 3B and C]. Consistent with previous reports, PD-0332991 promoted cancer cell migration and invasion. Although JQ1 has been considered a potential anticancer agent, it has also been shown to activate oncogenic genes or pathways that ultimately facilitate tumor progression^[23,41]^. In our study, JQ1 monotherapy likewise induced cell invasion and upregulated the expression of oncogenic markers such as CDH1, CDH2, VIM, and SNAI2 [Figure 8A and B, Figure 9A and B]. However, combined treatment with JQ1 and PD-0332991 significantly reduced tumor cell migration and invasion in 70301 and 60400 cell lines [Figure 3F].

### BET inhibitor JQ1 combined with PD-0332991 synergically suppresses EMT in PADC

In this experiment, we aimed to investigate the mechanism by which JQ1 suppresses EMT in PDAC. Western blotting results showed that treatment with 1 μM PD-0332991 increased N-cadherin levels while decreasing E-cadherin levels in 60400 cells [Figure 9C] and 70301 cells [Figure 8C]. Similarly, treatment with 0.5 μM JQ1 also upregulated N-cadherin expression and downregulated E-cadherin expression. Notably, the combined treatment significantly increased the protein levels of active (non-phosphorylated) β-catenin in both 60400 [Figure 9C] and 70301 cells [Figure 8C].

### BET inhibitor JQ1 partially rescues PD-0332991-induced EMT via Wnt/β-catenin activation

Western blotting further demonstrated that although E-cadherin expression was reduced, the addition of JQ1 suppressed the levels of N-cadherin, phosphorylated GSK3β(Ser9), phosphorylated Smad3, and active β-catenin. These findings suggest that JQ1 attenuates PD-0332991-induced TGFβ/Smad and Wnt/β-catenin cross-talk by modulating the phosphorylation status of GSK3β in 70301 cells [Figure 8C and D] and similarly in 60400 cells [Figure 9C and D]. Collectively, these results indicate that JQ1 can partially counteract PD-0332991-induced EMT through regulation of the Wnt/β-catenin pathway and exerts a synergistic inhibitory effect.

### A combination of BET and CDK4/6 inhibitors suppresses PDAC tumor growth *in vivo*

**Figure 10:**
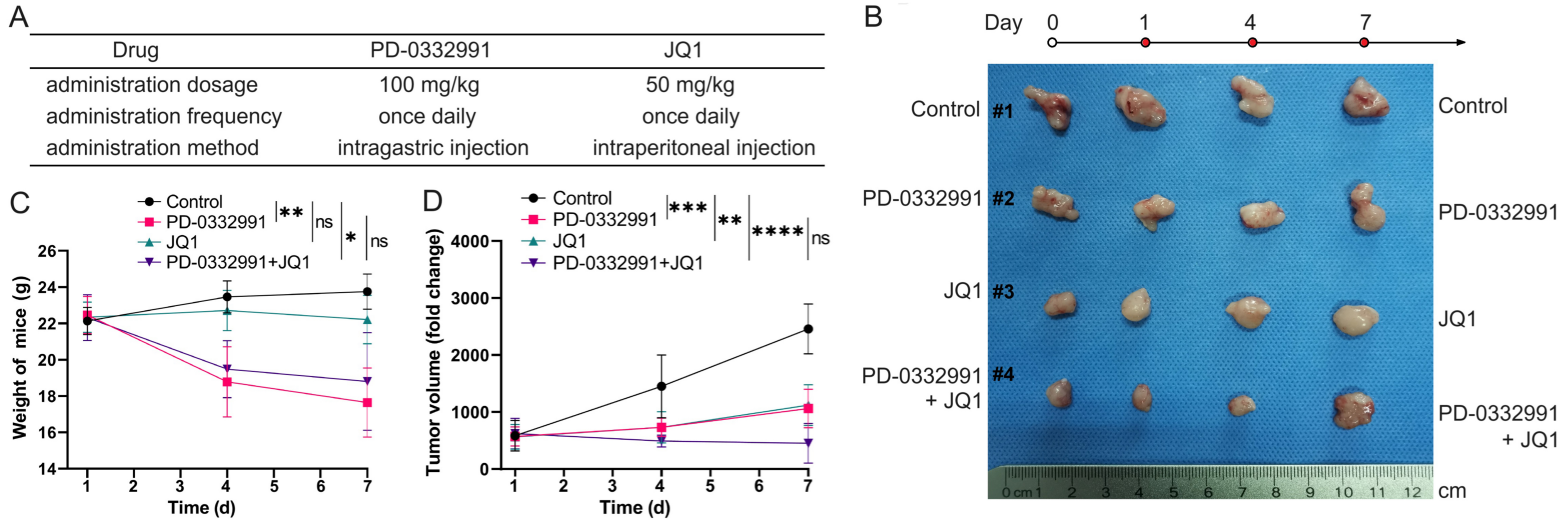
Combined PD-0332991 and JQ1 inhibited tumor growth *in vivo*. (A) Treatment regimens used in the *in vivo* study; (B) Xenograft tumors harvested from different groups of nude mice; (C) Body weights of mice in each group; (D) Tumor volume changes in response to treatment. Data are presented as mean ± SEM from three independent experiments. ^*^P < 0.05, ^**^P < 0.01, ^***^P < 0.001, ^****^P < 0.0001; ns: not significant. SEM: Standard error of the mean.

To evaluate the combinational effect *in vivo*, 70301 cells were subcutaneously injected into nude mice. Once tumors reached approximately 1 cm^3^ in volume, the mice were randomly assigned to different treatment groups. The treatment doses, frequency, and duration are illustrated in Figure 10A. The *in vivo* findings were consistent with the *in vitro* results*.* Tumor volume and body weight measurements are shown in Figure 10B. We found that monotherapy with PD-0332991 produced only a modest effect, whereas the combination of PD-0332991 and JQ1 elicited a marked therapeutic response without evident additional cytotoxicity, as indicated by stable body weights [Figure 10C and D].

### Combination of BET and CDK4/6 inhibitors suppresses the EMT in PADC *in vivo*

**Figure 11:**
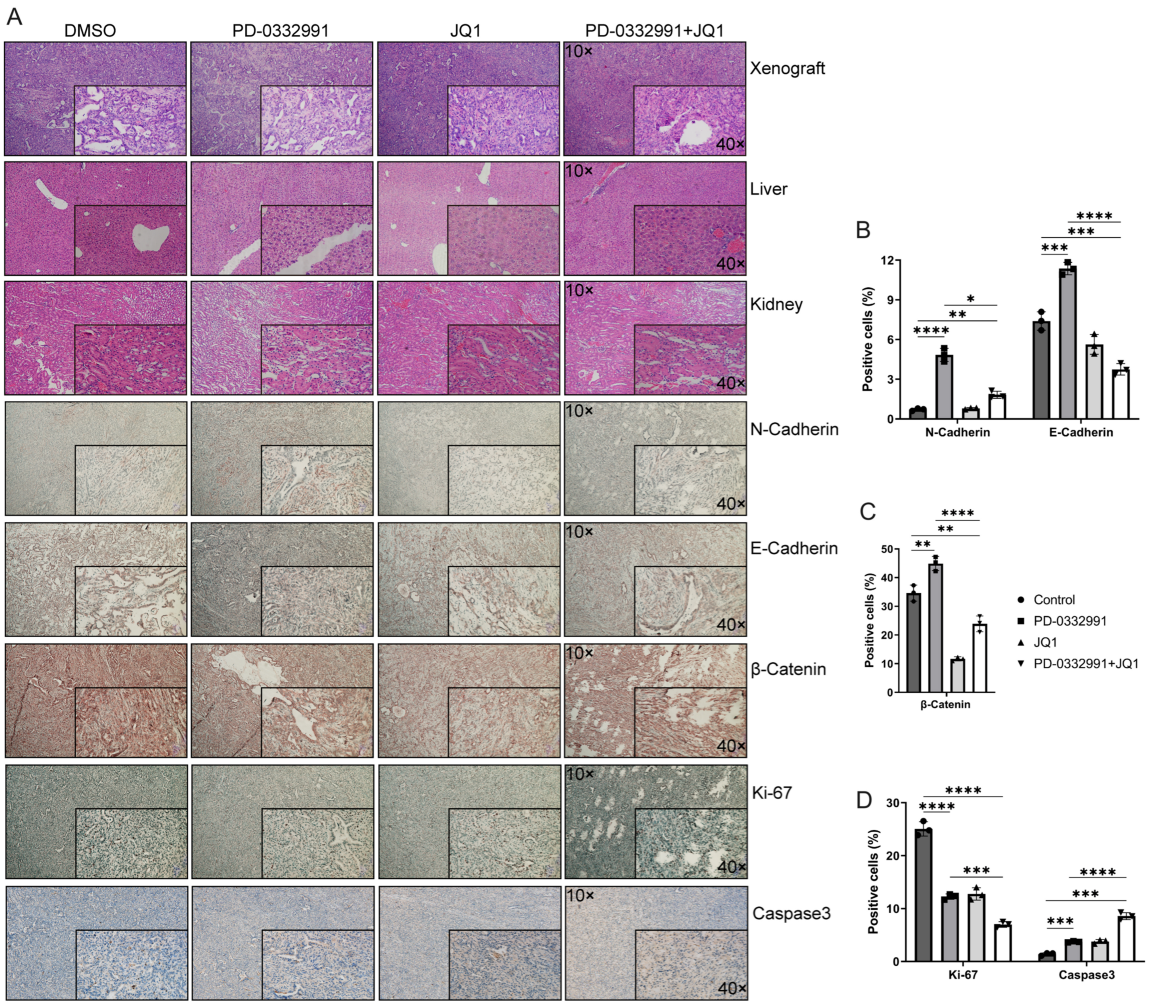
Morphological evaluation of xenograft tumors and liver and kidney tissues in nude mice using HE staining and IHC. (A) Nude mice were treated with PD-0332991 and JQ1. Primary antibodies against E-cadherin, N-cadherin, Ki-67, caspase-3, and active (non-phosphorylated) β-catenin were used for IHC analysis of xenograft tissues. Scale bars, 100 µm, 50 µm; (B-D) Quantitative analysis of E-cadherin, N-cadherin, active (non-phosphorylated) β-catenin, Ki-67, and Caspase-3 expression levels in xenograft tissues. Data are presented as mean ± SEM from three independent experiments. ^*^P < 0.05, ^**^P < 0.01, ^***^P < 0.001, ^****^P < 0.0001; ns: not significant. HE: Hematoxylin and eosin; IHC: immunohistochemistry; SEM: standard error of the mean.

**Figure 12:**
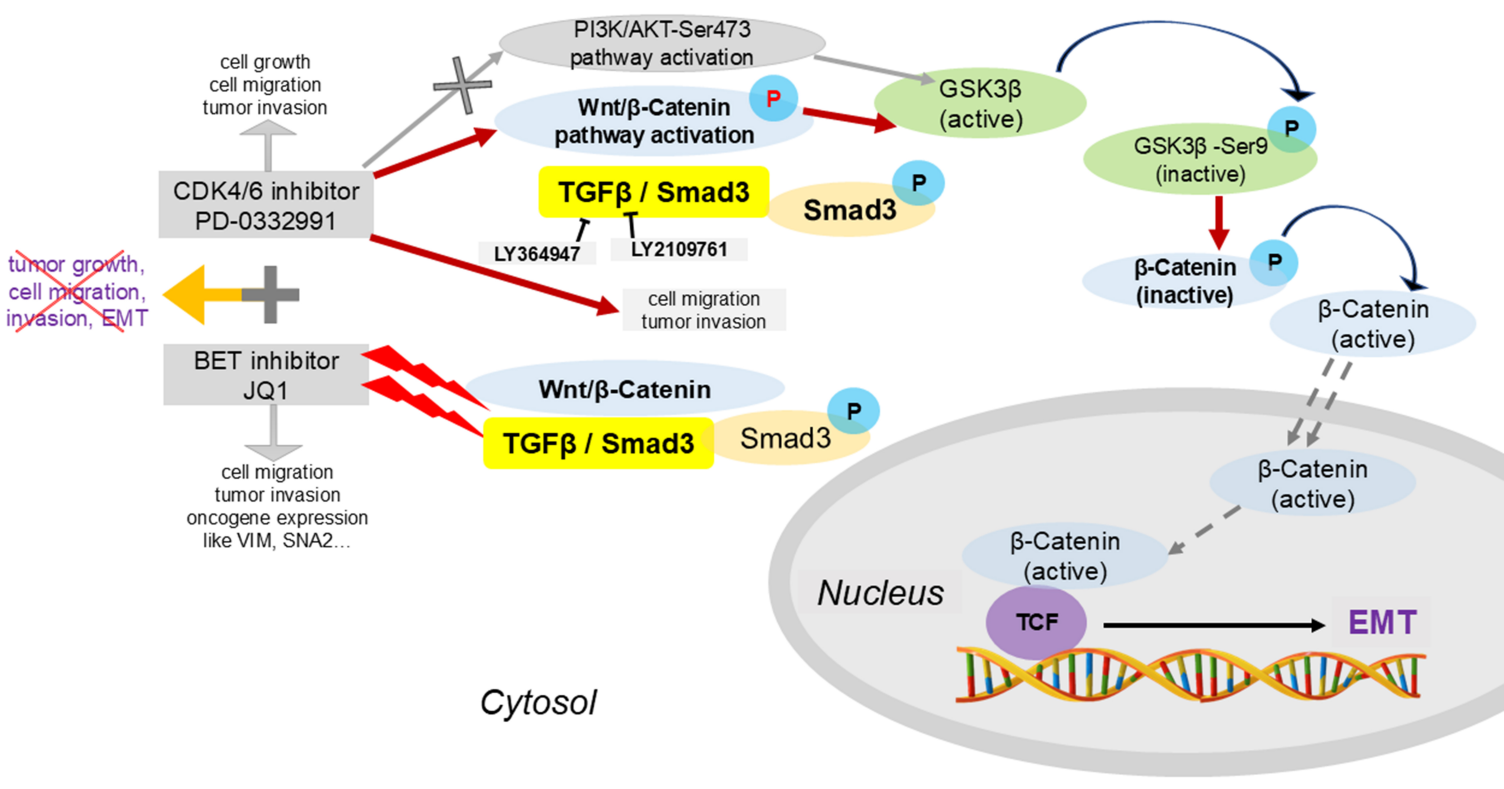
Schematic illustration of the potential mechanisms underlying combined BET and CDK4/6 inhibition. Palbociclib (PD-0332991), a CDK4/6 inhibitor, regulates GSK3β phosphorylation and activates the Wnt/β-catenin pathway, thereby promoting EMT through the TGFβ/Smad signaling pathway. This process is inhibited by the BET inhibitor JQ1. The combination of PD-0332991 and JQ1 may suppress pancreatic tumor cell growth, migration, invasion, and EMT. BET: Bromodomain and extra-terminal; CDK4/6: cyclin-dependent kinases 4 and 6; EMT: epithelial-to-mesenchymal transition.

Hematoxylin and eosin (HE) staining revealed no morphological abnormalities or tissue damage in the liver or kidneys [Figure 11A]. IHC analysis of xenograft tumors demonstrated that, compared to the untreated control, N-cadherin expression was markedly upregulated following PD-0332991 monotherapy, remained largely unchanged with JQ1 monotherapy, and was only slightly increased with the combination of PD-0332991 and JQ1 [Figure 11B]. Notably, E-cadherin expression was upregulated by PD-0332991 monotherapy, significantly downregulated by JQ1 monotherapy, and further reduced under the combination treatment [Figure 11B]. Active β-catenin levels were reduced following both JQ1 monotherapy and the combination therapy [Figure 11C]. For proliferation and apoptosis markers, Ki-67 expression was reduced, while caspase-3 expression was elevated in response to JQ1 monotherapy and combination therapy [Figure 11D]. These results suggested that the combination of PD-0332991 and JQ1 may inhibit tumor growth without inducing additional toxicity *in vivo*. The synergistic effects of PD-0332991 and JQ1 on PADC are summarized in Figure 12.

## DISCUSSION

A high frequency of loss-of-function mutations in CDKN2A and elevated CDK4/6 expression have been reported in PDAC, suggesting that CDK4/6 inhibition may offer therapeutic benefit. However, both preclinical studies and clinical trials have shown limited efficacy of CDK4/6 inhibitor monotherapy in breast and pancreatic cancers^[15,42]^. For instance, results from the TAPUR Study (ASCO 2019) indicated that PD-0332991 monotherapy exhibited no clinical activity in patients with advanced biliary or pancreatic cancers harboring CDKN2A loss or mutation^[43]^. While CDK4/6 inhibitors can arrest tumor cells in G1 phase and block mitosis, they may also antagonize the effects of conventional chemotherapeutic agents such as gemcitabine and fluorouracil^[44]^. Notably, sequential administration of PD-0332991 with taxanes (cytotoxic chemotherapeutics) has shown synergistic suppression of tumor growth *in vitro* and *in vivo*, underscoring the importance of treatment scheduling and rational combination strategies^[45]^.

GSK3β has emerged as a potential therapeutic target in cancer because it lies at the crossroads of several key signaling pathways, including Wnt/β-catenin, RAS/RAF/MEK/ERK, PI3K-AKT, and AMPK signaling pathways. The dynamic balance between phosphorylation by kinases such as GSK3β and dephosphorylation by phosphatases is important for regulating these pathways^[46]^. Activation of PI3K-AKT signaling induces AKT phosphorylation at Ser473, which in turn can phosphorylate and inactivate GSK3βat Ser9. Inactive p-GSK3βtriggers p-β-catenin dephosphorylation, promoting its activation and nuclear translocation - this constitutes the canonical mechanism through which GSK3β phosphorylation at Ser9 activates the Wnt/β-catenin pathway^[47-49]^. Importantly, Wnt/β-catenin activation is one of the most common mechanisms underlying drug resistance in cancer therapy.

In this study, we found that the FDA-approved CDK4/6 inhibitor palbociclib (PD-0332991) induced GSK3β phosphorylation at Ser9 via the TGFβ/Smad pathway, thereby activating canonical Wnt/β-catenin signaling. These changes may contribute to EMT induction and tumor cell migration. Thus, PD-0332991 monotherapy not only exerted limited growth inhibition but also activated oncogenic signaling. In contrast, combining PD-0332991 with the BET inhibitor JQ1 attenuated these effects *in vitro* and suppressed tumor growth *in vivo* [Figure 12]. Interestingly, JQ1 monotherapy, like PD-0332991, promoted PDAC cell migration and invasion and upregulated EMT-related genes in a dose-dependent manner, although the cells remained relatively sensitive to treatment.

Given the limited efficacy and potential oncogenic signaling triggered by CDK4/6 inhibition in PDAC, we screened our drug library and identified JQ1 and PD-0332991 as a synergistic combination. Strikingly, their combination not only enhanced tumor growth inhibition both *in vitro* and *in vivo* but also mitigated the oncogenic side effects observed with monotherapy. Mechanistically, JQ1 inhibited GSK3β phosphorylation and suppressed EMT by downregulating TGFβ/Smad signaling through decreased p-Smad3 expression [Figure 12]. However, the precise molecular basis by which JQ1 modulates the TGFβ/Smad pathway and its role in epigenetic regulation in pancreatic cancer require further investigation.

Consistent with previous reports, mutations in CDKN2A were detected in over 70% of PDAC cases in this study. Theoretically, CDK4/6 inhibition should therefore be an effective strategy. However, we observe that PD-0332991 only moderately suppresses tumor growth while simultaneously promoting migration, invasion, and EMT. Importantly, the BET inhibitor JQ1 significantly enhanced PD-0332991-mediated tumor suppression while attenuating or even partially reversing the EMT. These results revealed that JQ1 can overcome the intrinsic limitations of PD-0332991 monotherapy in PDAC.

We further found that PD-0332991 activated Wnt/β-catenin signaling by regulating GSK3β phosphorylation at Ser9, a process critical for maintaining stem cell properties and self-renewal. Notably, PD-0332991 did not increase p-AKT(S473), and inhibition of PI3Kα/δ or AKT did not reduce GSK3β Ser9 phosphorylation. These results indicate that the PI3K–AKT pathway does not mediate the PD-0332991–induced activation of GSK3β–Wnt/β-catenin signaling or EMT. Instead, PD-0332991 treatment led to upregulation of p-Smad3, linking its effects to the TGFβ/Smad pathway. Blocking this pathway in PDAC cell lines suppressed both EMT and GSK3β phosphorylation.

Although our study highlights a novel combination strategy in preclinical models of PDAC, several limitations remain. Tumor progression and metastasis are highly complex processes involving multiple genes and signaling pathways. Therefore, targeting one or even several pathways may not fully eradicate tumors. Moreover, compensatory oncogenic signaling or secondary mutations may emerge during or after treatment. While these challenges complicate therapy, they may also create opportunities for new targeted interventions. The therapeutic potential of targeting CDKN2A, especially in tumors with high mutation burdens, requires further exploration. Additionally, whether combining CDK4/6 inhibitors with agents such as MEK or PARP inhibitors could prevent EMT in PDAC remains speculative and warrants further investigation. Importantly, our findings are largely based on *in vitro* and *in vivo* models; thus, clinical trials and patient-derived data will be essential to establish translational relevance.

In conclusion, CDK4/6 expression is upregulated in PDAC and negatively correlated with patient survival. We developed a nomogram model incorporating CDK4/6 expression and clinical parameters to predict survival outcomes. Monotherapy with the CDK4/6 inhibitor PD-0332991 yielded limited therapeutic effects and paradoxically activated oncogenic Wnt/β-catenin signaling via TGFβ/Smad-mediated GSK3β phosphorylation, thereby promoting EMT. In contrast, combination therapy with PD-0332991 and the BET inhibitor JQ1 synergistically suppressed tumor growth both *in vitro* and *in vivo* and blocked EMT by inhibiting TGFβ/Smad-GSK3β-Wnt/β-catenin signaling. These findings provide a strong rationale for clinical development of combined CDK4/6 and BET inhibition strategies in PDAC and elucidate the underlying mechanisms of their synergistic effects in primary pancreatic cancer cells.

## DECLARATIONS

### Availability of data and materials

The raw data supporting the conclusions of this article will be made available by the corresponding authors upon reasonable request.

### Copyright

© The Author(s) 2025.
